# Inclusion and exclusion in the globalisation of genomics; the case of rare genetic disease in Brazil

**DOI:** 10.1080/13648470.2017.1381230

**Published:** 2018-03-13

**Authors:** Sahra Gibbon, Waleska Aureliano

**Affiliations:** a Department of Anthropology, University College London, London, UK; b Department of Anthropology, Universidade do Estado do Rio de Janeiro, Rio de Janeiro, Brazil

**Keywords:** Brazil, genetics, rare disease, inclusion/exclusion

## Abstract

Within the context of a globalising agenda for genetic research where ‘global health’ is increasingly seen as necessarily informed by and having to account for genomics, the focus on rare genetic diseases is becoming prominent. Drawing from ethnographic research carried out separately by both authors in Brazil, this paper examines how an emerging focus on two different arenas of rare genetic disease, cancer genetics and a class of degenerative neurological diseases known as Ataxias, is subject to and a product of the dynamics of inclusion and exclusion as this concerns participation in research and access to health care. It examines how in these different cases ‘rarenesss’ has been diversely situated and differently politicised and how clinicians, patients and their families grapple with the slippery boundaries between research, rights to health and the limits of care, therapy or prevention. It illustrates how attention to rare genetic disease in Brazil emerges at the intersection of a particular history of genetic research and public health infrastructure, densely complicated feedback loops between clinical care and research, patient mobilisation around the ‘judicialisation’ of health and recent state legislation regarding rare disease in Brazil. It highlights the relevance of local configurations in the way rare genetic disease is being made relevant for and by different communities.

## INTRODUCTION

Definitions of rare disease are internationally variable, often defined in terms of prevalence and sometimes further qualified with reference to conditions that are ‘life threatening’ or ‘chronically debilitating’. WHO figures suggest that 8% of the world's population are affected by rare genetic disease and there are between 6000 and 7000 rare diseases worldwide. With 80% of rare diseases thought to be genetic in origin and many related to single gene disorders, a focus on rare disease has long been part of genetic medicine. This is now being extended across national and transnational terrains of health care and research. This is evident as much in the expanded scope for newborn genetic screening in the US and the UK aimed at including a wider range of rare potentially disabling conditions (Timmermans and Buchbinder 2012) as it is in high-profile developments such as the UK's 100,000 Genomes Project. In both cases, rare genetic disease is explicitly described as the ‘ideal platform for translational medicine and the application of high throughput genomics in routine NHS practice’ (Strategic Priorities Working Group for the 100K Genome Project 2013).^1.^ At the same time, a focus on rare genetic disease variants is considered important in efforts to extend the global diversity of genomic research (Bustamente, De la Vega, and Burchard 2011).^2.^

The role of patient organisations has also been central in reframing the social and political significance of rare genetic disease and in some cases the scientific trajectory of research, particularly in Europe and North America (Rabeharisoa and Callon 2004). While the ‘activism’ at stake in patient organisations is varied (Epstein 2008), it has, in some cases, resulted in the productive alignment between rare disease and patient exclusion. This has transformed ‘uncommon disorders’ into ‘rare diseases’ (Huyard 2009) and lead to changes in the regulation of clinical trials involving so called ‘orphan drugs and diseases’ in the US and Europe.^3.^ However, as Rabeharisoa et al. (2014) point out, various strategies of politicisation can be identified within diverse patient organisations focused on rare and genetic disease with consequences for the scope and limits of research and resources across different national arenas, particularly in the US and Europe.

Drawing from ethnographic research carried out separately by both authors in Brazil, a neglected region outside of Europe and North America, we show how an emerging focus on two different arenas of rare genetic disease is subject to and a product of the dynamics of inclusion and exclusion as this concerns participation in research and access to health care. Sahra Gibbon carried out fieldwork in three different cancer genetic clinics in urban regions of Brazil in the southern part of the country between 2010 and 2012. This included observations of clinical consultations and semi-structured interviews with patients, health professionals and scientists. Waleska Aureliano carried out ethnographic research in Rio de Janeiro, between 2012 and 2014. This included working with patients and families linked to an Ataxia patients’ organisation, observing appointments in the genetic service of a public hospital and formal meetings to discuss public policies related to rare diseases.^4.^

This article illustrates how attention to rare genetic disease in Brazil emerges at the intersection of specific socio-historical and cultural dynamics. This includes a particular history of genetic research and public health infrastructure, densely complicated feedback loops between clinical care and research, patient mobilisation around the ‘judicialisation’ of health and recent state legislation regarding rare disease in Brazil.^5.^ In examining these intersections, we make a significant contribution to an evolving field of social science research, highlighting the relevance of local configurations in the way rare genetic disease is being made relevant for and by different communities. First, we outline how interest in rare genetic disease has emerged within genetic medicine in Brazil, in dialogue with paediatrics and oncology and in relation to a partial and inequitably distributed system of public health. This has led to entanglements between research and clinical care resulting in, but also made more complex by, the growing judicial demands for treatment from patients as well as a recent national strategy to integrate genetics into public health. We then draw on ethnographic research in Brazil examining two contrasting conditions in which ‘rarenesss’ has been diversely situated and differently politicised: cancer genetics and a class of degenerative neurological diseases known as Ataxias. In each of these cases, clinicians, patients and their families grapple with the slippery boundaries between research and rights to health as well as the necessity and limits of care, therapy and prevention. Collectively, these reflect and reproduce specific dynamics of inclusion and exclusion. We demonstrate how these play out differently, even paradoxically, across and within specific arenas of genetic medicine and how research and medical practice is dynamically constituted by and helps shape the meaning of ‘rareness’. In the Brazilian contexts we have examined, ‘rare disease’ is a flexible categorisation that is made both mutable and mobile; sometimes it is explicitly foregrounded or alternately it exists in tension with a discourse of disability or cancer as a common disease. In examining the situated meanings and strategic mobilisations of rareness, we underline the central importance in Brazil of ‘biolegitimacy’ (Fassin 2009) and ‘rights’ to health care in the globalisation of genomic medicine.

## Genomics and rare genetic diseases in Brazil

In Brazil, medical genetics has only been recognised as a clinical and research specialty since 1983. In 2013, the Federal Council of Medicine registered 200 medical geneticists for the entire country, with 80% of them working in the south and southeast regions and with four Brazilian states in the north of the country with no medical genetic services (Scheffer 2013).

While the number of private genetic testing companies, particularly for paternity testing, is expanding in Brazil, the majority of clinical services in medical genetics are concentrated in university or public hospitals within large urban centres or in reference centres for research and/or blood donation (Melo et al. 2015; Horovitz et al. 2013). Somewhat paradoxically, however, medical genetics in Brazil is not a domain of expertise that is fully integrated into the public health care system, known as the *Sistema Único de Saúde* (SUS) with the position of a medical geneticist not ‘officially’ recognised (Vieira et al. 2013). These professionals are usually based in university hospitals and research centres with virtually none currently working in the primary health care. This situation is particularly relevant in a context where the health system is de-centralised and hierarchical as it is in Brazil. As a result, patients are obliged to go to primary health care centres to obtain tertiary care services, such as genetic diagnosis and testing.

However, referral to the right medical specialist may be more complex as many doctors do not know or recognise most of the rare genetic diseases that exist in the country. This occurs particularly (but not exclusively) in people with late onset rare diseases. Currently, the only way of mapping people suffering from genetic diseases is what is known as a liveborn declaration or *declaração de nascido vivo’* an official document issued by hospitals, without which a child's parents cannot register the birth of the child. While this certificate records information about congenital abnormalities, the registry does not record people that inherit genetic disease associated with adult onset conditions, such as Huntington Disease or Machado–Joseph Disease (MJD), or even early onset genetic diseases, but which may not be evident at birth.

Despite a scarcity both in terms of reference centres and professionals with expertise or formal knowledge in medical genetics, since the 1990 s, there have been a number of genetic health programmes. This includes the National Newborn Screening Program (*Programa Nacional de Triagem Neonatal—*PNTN) created in 2001 that tests all newborns for four diseases: phenylketonuria, congenital hypothyroidism, cystic fibrosis and sickle cell disease and other hemoglobinopathies. The development of this programme led to the establishment of national testing centres.^6.^

Two medical fields are especially important in considering how efforts at incorporating genetics into *SUS* have and are continuing to evolve: pediatrics and oncology. The first advocates wider coverage of the newborn screening programme, to include more diseases, and also the integration of genetic counselling into primary health care. The aim is to reduce the number of newborns with congenital anomalies that in the year 2000 was the second cause of death in children under one year of age in Brazil (Horovitz et al. 2013). In the case of oncology, cancer genetics has developed in Brazil over the last 10 years in deep and complex relation to research and public health services, as discussed below.

In Brazil, genetic research related to cancer and pediatrics, are directly connected to the national system of free and universal public health. For the most part, monetary and research investments for genetic diseases in Brazil comes from the public sector, especially the federal government, and also via networks of transnational research. Somewhat contradictorily, the State part funds research in the context of a clinical genetic service not only considered insufficient by doctors and patients but which is itself mostly sustained as a result of research funds. Until recently, only a few rare genetic diseases were included in SUS treatment protocols (among them Osteogenesis Imperfecta, Cystic Fibrosis and Gaucher Disease). Some drugs used to treat these conditions are included in the list of high-cost medicines approved by the National Health Surveillance Agency (*Agência Nacional de Vigilância Sanitária*—ANVISA). However, even in these cases many families affected by rare genetic diseases may need to initiate what are known as judicial demands to obtain the drugs.

## Judicialisation, research and public policies for rare diseases in Brazil

The ‘judicialisation’ of health has become a growing phenomenon in Brazil over the last 10 years, involving persons from a range of social classes (Biehl 2013). The constitutional commitment by the State to provide health care to all Brazilians citizens has led many families, individuals and sometimes also patient associations affected by rare diseases to seek the right to certain health care products and services, through legal tribunals. These claims range from access to drugs not approved by ANVISA, to high-cost medicines not included in SUS lists, to increasingly genetic tests and also in some cases the ‘right’ to take part in experimental clinical trials (Castro 2015; Diniz, Medeiros, and Schwartz 2012).

Despite financial and structural limitations, Brazilian patient associations have been active and highly influential in political lobbying resulting in the construction of public policies directed at people living with rare diseases. However, it is important to note how in contrast with many Euro-American patients’ associations (Huyard 2009; Rabeharisoa et al. 2014), Brazilians groups are not necessarily motivated to find a ‘cure’ for rare diseases. The focus of their actions is rather to ‘oblige’ the State to support and fund health care services for those with rare diseases and their families (Grudzinski 2013; Aureliano 2015). In many cases, this means access to high-cost medication. Since the 1990 s, after the creation of the Brazilian public health system, there have been several successful achievements, directly stemming from the participation of patients’ associations, to increase attention to rare or rather neglected diseases. For example, the current public health care policy for sickle cell disease (SC) was developed following strong lobbying by patients’ organisations and particularly the Brazilian *Movimento Negro* or Black Movement, due the historical link between SC and black populations (Silva 2014). As a result, SC is now one of the diseases tested by the national programme of newborn screening. A similar scenario occurred with Gaucher's disease, where drugs used to treat the condition were included in the National Exceptional Drugs Program following litigations pursued by affected families against the State.

In 2009, the Directive 81/09 from Ministry of Health established a National Policy of Comprehensive Care in Medical Genetics. The aim was to create public health programmes that offered genetic counselling and testing. This was, however, thwarted by operational and conceptual problems including the scarcity of health professionals with knowledge in medical genetics.

Analysing these developments, Novoa and Fróes Burnham (2011, 65) call attention to the relevance of distinguishing medical genetics that is ‘important and applicable to public health and that which is still object of research’. However, in practice, this separation has not proved easy or sustainable in Brazil since many research centres depend on biological material collected in the clinics. In turn, many patients depend on research to obtain a conclusive diagnosis for diseases without cure or treatment, which might nevertheless, for example, guarantee rights, such as accessing certain state benefits. Due to the lack of definition of norms regarding their medical utility and the difficulty of disentangling the aims of research and public health, genetic tests are seen as a great challenge for public policy. The situation is even more complex for asymptomatic subjects where knowledge of risk cannot always be directly linked to preventative health care treatments or interventions.

More recently in Brazil, the joint action of government, scientists, physicians and associations has led to the development of the *National Policy for Comprehensive Care of People with Rare Diseases (PNAIPDR)*, instituted through Directive 199, published in January 2014.^7.^ It provides a mandate for the creation of medical genetics referral services that include primary health care and which are equipped to provide specialised care for people with rare diseases. To achieve these goals, various actions and procedures will need to be created within the public health system, including expanding the list of medications offered and widening the scope of genetic testing and counselling. As some patient associations have already stated, the current challenge is to ensure the policy is fully enacted, something which did not happen in the case of Directive 81/09.

However, the *PNAIPDR* also envisages that it is the duty of the Ministry of Health to ensure the development of research that contributes to technological innovation and dissemination of knowledge related to promoting health, prevention, care and rehabilitation of people with rare diseases. This is an important issue for many medical genetic professionals who frequently work in and must negotiate the liminal space between clinical care and research. In this new scenario, many researchers and patient advocates argue that the same ethical principles and legislation for researching non-rare disease would make it almost impossible for Brazilian patients to take part in clinical trials sponsored by international laboratories.

An important illustration of these difficulties is provided by Castro's (2015) analysis of a public consultation undertaken in 2011 to review Resolution 196 of the National Council of Health that defines the ethical principles of research with human beings in Brazil. One of the stated norms of this resolution relates to the duty of research sponsors to provide for patient research subjects the best therapeutic, diagnostic and prophylactic methods identified in the clinical trials, for an undetermined time. This premise aims to reduce judicial claims against the State to access experimental drugs that are not yet approved by Brazilian health organisations.

Most of the 300 commentaries from the public consultation in response to this new regulation came from doctors, but also from patients and families affected by rare diseases. According to many of those who responded, the mandate to provide such services to patient subjects would reduce the probability of national and international commercial laboratories conducting clinical trial research with Brazilian patients with rare diseases. Freely supplying the drugs that resulted from patient involvement in research would, critics of the resolution argued, make commercial investment unfeasible and unappealing. As a result, many doctors and patients claim that specific ethical principles should be developed for clinical trials conducted with rare disease patients. Most striking in these arguments is the way that clinical trials are compared to treatment.^8.^

The concept of ‘rareness’ acts here as an important issue in these debates. Similar to the displacement realised by the pharmaceutical industry in Europe between profit and innovation in the context of rare diseases as examined by Huyard (2009), we can say that, in Brazil, there is a displacement between clinical research and treatment/health services in an effort to participate in global genomic research initiatives that meet international ethical standards of human subject research.^9.^ This has particular consequences for patient and health care provision especially when, as Castro (2015) points out, to be included in a clinical trial may be the only hope for those who have diseases for which there are currently no treatments or cures.

In the next section, we examine in more detail how some of these aspects are unfolding in relation to contrasting domains of attention to rare genetic disease in Brazil, cancer genetics and a particular class of neurodegenerative disorders know as Ataxias.^10.^

## Oncogenetica in Brazil; between common conditions and rare genetic syndromes

While the range of diseases addressed within Brazilian cancer genetics is broad, in this article we examine two specific avenues of inquiry. This includes the high-profile context of BRCA genes associated with breast cancer and a particular biomarker R337h located on the TP53 gene that has been identified in the south and southeast of Brazil and associated with what is normally thought to be a ‘rare’ cancer syndrome known as Li-Fraumeni. Cancer genetics is not currently included in the Government Directive to incorporate rare genetic disease into the public health. Nevertheless, examining these domains of Brazilian cancer genetics illuminates how what becomes part of the terrain of research and care is informed by strategic mobilisation of articulations of ‘rareness’. This is constituted as currently ‘unknown risk’ in the case of the BRCA genes or in terms of a disease which is both ‘rare’ *and* ‘common’ in the case of R337h. At the same time, there are also contrasting cultures of activism in these different areas of cancer research where ‘rights’ to health care are articulated in specific ways.

The specialism of cancer genetics has emerged in Brazil in the last 10–15 years linked to research and university hospitals predominantly, although not exclusively, in the relatively wealthy southern urban centres. While this regional and institutional location reflects differential national cancer incidence, it is also a product of and informs a system of ‘hidden innovation’ (Souza 2015) in which clinical interventions are entangled with and dependent on national and transnational research funding. The dense inter-relationship between research and the clinic has been described by others as a characteristic feature of the ‘clinical collectives’ that have emerged in ‘translational’ cancer genetics in many national contexts, where the boundary between clinical care, research and innovation is highly diffuse (Bourret 2005). In resource-poor contexts such as Brazil, dependency on national and transnational research investment has particular consequences for the way that an ethic of care (Gibbon 2015) and of ‘solidarity’ (Souza 2015) become folded into the pursuit of cancer genetics.

## The productive uncertainty of unknown (‘rare’) BRCA variants and common cancers

In Brazil, research focused on the two high-profile inherited susceptibility genes BRCA 1 and BRCA 2 has been a significant domain of activity, including efforts to identify potential founder mutations. Brazilian research examining internationally well characterised mutations on the BRCA genes has been directly facilitated by transnational collaborations examining how genetic ancestry may variably contribute to the high rates of cancer in the southern region of the country. It is a goal which co-exists alongside a desire to develop quick and cheap means of expanding the scope of genetic testing in Brazil in the context of public health concerns about the rising rates of cancer in the country (Gibbon 2015). Yet, common founder mutations linked to the BRCA genes have not been identified with any great frequency. It is a terrain of research and medical intervention which is as a result in Brazil (as elsewhere), characterised by a large amount of uncertainty with the frequent identification of unknown mutations and what are described as ‘Variants of Unknown Significance’ or VUS.

For many members of the cancer genetic community in Brazil, this is a situation of ‘unknowing’ that warranted further urgent investment in cancer genetics. There has been a strong lobbying group of health professionals linked to specialist cancer and university research hospitals, which have been campaigning for some time for the incorporation of cancer genetics within the *SUS* public health system. This has coalesced around networks such as a national Hereditary and Familial Cancer Network, which have consistently emphasised the ‘specificity’ of the Brazilian population (INCA 2009).

The identification of specific variants of unknown risk, while generating clinical uncertainty, can also facilitate particular avenues of research. For example, during fieldwork undertaken by Gibbon, the discovery of a variant on the BRCA genes normally associated in the scientific literature with ‘African American Ancestry’ caused a degree of unease and hesitancy among Brazilian researchers concerning how this should be communicated at the clinical interface to patients. At the same time, it also extended and generated novel transnational research collaborations and networks (Gibbon 2016). As Brazilian cancer genetic practitioners negotiate the issues concerning ‘missing heritability’, ‘under-served populations’ and also engage with the increasing stratification of cancer in translational research, their efforts to localise unknown variants on the BRCA genes as forms of ‘rare’ disease may yet prove a viable means of extending networks of research.

Yet, it is also important to note how the identification in Brazil of multiple variants on the BRCA genes of unknown or uncertain significance also have consequences beyond the specific disciplinary confines of cancer genetics. While productive research and clinical collaborations between oncology and genetic specialists may unfold as a result of these developments, it is not guaranteed. In one hospital included in research undertaken by Gibbon, those working in mastology (a clinical sub-discipline with an established history in Brazil) resisted efforts to include predictive genetic testing interventions in their clinical repertoire. This did not, in their view, contribute to the wider goals of addressing the high rates of breast cancer in Brazil. As one Brazilian mastologist put it,

If you don't have information about the criteria for risk assessment in Brazil, you're going to start wasting money and we don't really have money to waste here, even more so in the public health system!

In a resource-limited public health system, where addressing basic health care issues related to ‘common’ diseases such as breast cancer are a significant challenge, these comments underline differences in responses to genetic interventions. This raises further questions about what kinds of ‘bio-clinical collectives’ (Bourret 2005) can coalesce around genomic knowledge and the extent to which attention to ‘rare’ or unknown genetic variants associated with cancer risk limit or mobilise different medical specialities.

## R337h, ‘rare’ cancer syndromes; specificity as inclusion

Another prominent domain of inquiry within Brazilian cancer genetics has focused on a particular variant described as R337h, located on the TP53 gene. Aligning and extending particular kinds of national and transnational research collectives, this is an arena of research where engagement with (and contestation of) ‘rareness’ has been both highly visible and productive.

Common germline mutations on the TP53 gene are infrequent, estimated in the US to be around 1 in 5000 but which are associated with a rare cancer syndrome known as Li-Fraumeni that has been linked to an up to 90% lifetime chance of developing different types of cancer, particularly in children (Malkin et al. 1990). Brazilian studies in the early 2000s suggested that a specific variant R337h located on the TP53 gene is particularly frequent in the south of the country, with a purported prevalence of 1 in 300 in these regions. It has been initially associated with a range of rare childhood cancers and subsequently with more common adult cancers, including breast cancer (Giacomazzi et al. 2014).

The prevalence of the mutation in the southern region of the country has been of significant interest for members of the cancer genetic and paediatric community in their effort to constitute this as a significant public health issue and coalesce national and transnational research interests. The attempt to define the nosology of the syndrome has also entailed shifts between an association *and* contrast with the normally ‘rare’ syndrome Li-Fraumeni and more common cancers such as breast cancer. Moreover, while heated debates continue about the association of R337h with adrenocortical cancers in children and/or other common cancers in adults (see Achatz, Hainaut, and Ashton-Prolla 2009), innovation and investment have continued in this field. This includes efforts in 2013 to develop a cheap and rapid testing technology for mass screening of R337h (Arruda and Sensato 2013) and evolving epigenetic research which locates R337h in wider programmes of international research in Brazil concerned with metabolism and cancer (Armstrong 2015). Like the terrain of BRCA genetics, research on R337h is also subject to uncertainty which has nevertheless become part of an extended and broad terrain of research inside and outside Brazil. In the case of R337h, this has successfully aligned diverse specialisms including cancer genetics, paediatrics and now increasingly epigenetic research. Significantly, many members of the clinical and research teams involved in the Brazilian cancer genetics community commented that it was research related to R337h, rather than the high-profile terrain of BRCA genetics, which they felt was most likely to facilitate the expansion and consolidation of cancer genetics as a medical speciality in Brazil. This was how one member of a research team in Sao Paulo put it,

I think it (R337h) will be a major issue here in Brazil, because it is very frequent, much more frequent than BRCA mutations and the problem is that BRCA testing is also much more expensive than TP53. The thing is we have a pathway and a possibility for doing R337h. Whenever I have a patients with this diagnosis, I can see right away I can collect the sample, test it and right away give that patient a result.

While the BRCA genes have been directly associated globally with the high incidence of common cancers such as breast and ovarian cancer, which are rising at alarming rates in Brazil, ‘rareness’ is nonetheless implicitly highlighted in relation to what are ‘unknown’ genetic variants. By contrast, in relation to the normally ‘rare’ cancer syndrome Li-Fraumeni, the ‘specificity’ of the syndrome in Brazil is explicitly foregrounded and has successfully aligned diverse medical specialists. At the same time, the rareness of the syndrome in Brazil is directly contested, with an emphasis on the national and regional frequency of R337h, described as a ‘conditional cancer pre-disposing mutation’. Therefore, in the case of BRCA genes and R337h there is a productive movement and juxtaposition between the frequency and rareness of gene variants, diseases and syndromes that reflect and constitute specific avenues of inclusion and exclusion.

## Cultures of activism, ‘under-served’ populations and judicial rights to genetic testing

The cultures of activism around articulations of ‘rights to health care’, that have emerged somewhat differently in the context of research and medical interventions related to BRCA and R337h, provide another illustration of how diverse aspects of cancer genetics in Brazil are subject to different dynamics of inclusion/exclusion.

The way that cancer genetics operates at the interface between research and public health has particular consequences for how patients and others are ‘active’ in pursuing rights to health care interventions. Observations in clinical contexts illuminated how in the case of BRCA genetic testing a handful of patients and families in Sao Paulo, had pooled finances to be able to pay for a genetic test or were negotiating with their private health insurer to secure rights to screening and testing.^11.^ By contrast, there was a striking absence of this kind of ‘activism’ among so called ‘R337h patients.’ Many of those recruited into this particular research terrain were SUS patients without private health insurance and often very little access to basic health care facilities, particularly those families who came from the interior rural regions.

Nevertheless, a different sort of activism was manifest in this arena. This was nurtured and pursued by health professionals in a context where research has become entangled with an effort to provide what is seen as ‘preventative’ public health care for patients who were currently ‘under-served’. One particular manifestation of such activism was evident in what were described by researchers as ‘road trips’ undertaken by clinical and research teams to rural parts of the country to meet large extended families affected by cancer linked to the R337h mutation.

In one ‘road trip’, that Gibbon participated in, a visit to a family home in the rural area of the state of Sao Paulo had been facilitated by a young female patient who had been treated for cancer and also identified as having the R337h mutation. One Saturday morning in 2011, an array of relatives squeezed into the small sitting room of the patient's aunt to hear the information about the research from the lead practitioner. After explaining the details of the research where the specificity (if not explicitly the rareness) of the syndrome Li-Fraumeni in the south of the region was emphasised, and discussing consent with those who wanted to participate, over 20 samples were collected from the large extended family. The excitement that ensued for the clinical team in being able to calibrate and extend research related to R337h, was also however deeply entangled with their sense that incorporating extended family members into research was a means of providing neglected preventative health for ‘under-served’ communities. The only ‘care’ that could be offered to relatives was mostly dependent on identifying the presence of deleterious variants. However, the promise of future health provision, including, for instance, routine mammography screening within a highly regarded specialist cancer hospital, was often a sufficient justification and reason to participate for many family members.^12.^ In these situations, the gratitude of patients in being part of research initiatives was common. As one patient put it, in addressing the researchers during another community event that aligned affected families, care and research, ‘you are doing so much for us, what can we do for you?’.

While the ‘activism’ of practitioners in Brazilian cancer genetics seems to directly inform who and what gets encompassed within terrains of research and medical care, the expanding scope of judicialisation, in conjunction with the complex dynamics of research and health care in Brazil, is further shaping these dynamics.

In 2013, during a return fieldwork trip, Brazilian clinicians working in both public and mixed public/private hospital talked about the upsurge in judicialisation cases linked to rights to obtain genetic testing for the BRCA genes. Most directly commented that they felt this was as a result of the announcement by the actress Angelina Jolie a few months previously that she had had a BRCA test and undergone a prophylactic mastectomy. The discourse of ‘rights’ to testing, that had ensued at this time in the international media and press, was pervasive in Brazil and elsewhere leading to a national and indeed international increase in referrals to cancer genetic clinics (Evans et al. 2014). One clinician commented that of the 30 or so patients she had seen each week in the clinics at least one was now pursuing the judicial right to have a genetic test for the BRCA genes.

With currently no high profile-celebrity endorsing the ‘right’ to genetic testing and given the dearth and complexity of ‘preventative’ and prophylactic treatment interventions for a variant that is thought to lead to a multitude of cancers, it is not surprising to find that there are as yet very few judicialisation cases related to R337h. Yet, as the extensive ‘bioclinical collective’ (Bourret et al. 2005) which has emerged around the ‘common’ biomarker R337h associated with the ‘rare’ syndrome Li-Fraumeni evolves, and as the emerging horizon of different therapeutic and/or preventative screening interventions becomes clearer, this situation may change. Thousands of cases of judicialisation in Brazil are being processed by the courts not only for therapeutic treatments for rare genetic disease but also now for testing and screening for more common conditions. It seems likely that this will increasingly be an avenue by which those currently excluded from health care services, such as the significant and large numbers of Brazilians who are carriers of R337h, will seek to change and inform the parameters in which their rights to screening, testing and monitoring may be included.

In summary, even though cancer genetics is excluded from the recent Brazilian Government Directive to incorporate rare genetic disease into the public health system, different sorts of ‘activism’ and participation by researchers and patients inform how cancer genetics is nonetheless subject to specific dynamics of inclusion (and exclusion) related to research and health care access.

## From ‘rareness’ to ‘disability’, and vice-versa: the case of Ataxias

Ataxia is the name given to a series of degenerative neurological diseases that mainly affect movement and speech. Though various acquired forms of the disease exist, the majority are genetic and hereditary in origin. Machado-Joseph Disease is one of the inherited forms of Ataxia and was first identified in the medical literature in the 1970s, although it was only in the 1990s that it was genetically characterised. Initially, the disease was reported in North American families that were descended from Azoreans. While the prevalence of the condition in Azores’ Islands is very high, current studies suggest that the genetic mutation linked to MJD is present in other ethnic groups (Lopes-Cendes et al. 1997). It is an autosomal dominant inherited disease, which means that the probability of transmission to descendants is 50%. Generally speaking, MJD manifests in adult life around the age of 40, with principal symptoms including loss of balance, muscular paralysis, speech problems, difficulty in swallowing and double vision. Affected people can live for decades while the disease evolves before dying mainly from secondary complications. There is no curative treatment for MJD. Therapeutic interventions like physiotherapy and speech therapy aim to minimise the effects of the disease, which eventually leads to bodily paralysis.

In Brazil, this is the most frequent form of hereditary Ataxia, with a high prevalence in the south of the country, associated with Azorean immigration to this region. In the southern state of Rio Grande do Sul (RS), MJD represents 78% of all inherited Ataxias registered in this state (Saute and Jardim 2015). RS has a high concentration of families affected by MDJ, with a prevalence of 3 people to every 100,000 inhabitants. (Trott et al. 2006). It is in this state that the main Brazilian medical genetic laboratories and research groups are located, with 37 of the 50 physicians with specialisation in medical genetics based in RS (Scheffer 2013, 249). This regional focus that combines research about and care for those with MJD has stimulated further discussion about the complex intersections between research agendas and clinical care.

The Porto Alegre Polyclinic Hospital, for example, conducts tests for patients from a number of Brazilian states, employing resources and professional staff linked to a research project run by the Federal University of Rio Grande do Sul (UFRGS). The project's main objective is to make the molecular investigation of neurogenetic conditions available for individuals from diverse parts of Brazil, forming a national research network on such diseases. Today, 11 hospitals are linked to this network,^13.^ through which many people with symptoms of inherited Ataxias can get a diagnosis for their diseases.

Rio Grande do Sul is also the location for INAGEMP (The National Institute of Medical Population Genetics) that is part of the National Institute of Science and Technology created in 2008 by the federal government. Such institutes aim to develop innovative research and produce national patents, connecting research centres from different regions. The network created by INAGEMP includes some groups dedicated to investigating MJD^14.^ in partnerships with Portuguese researchers since Portugal and the Azores have a high incidence of MJD, and because colonisation is considered a central factor in the development of the disease in Brazil.

Brazilian research on MJD investigates a range of aspects, including molecular dimensions and phenotypic variability (Saute and Jardim 2015), the implications of predictive tests for asymptomatic individuals from affected families and the need for genetic counselling (Schuler-Faccini et al. 2014). Studies on medications to control or delay the course of the disease (Monte et al. 2003; Saute et al. 2014) have not yet observed improvements in patients’ health, despite increased knowledge of hereditary Ataxias.

Studies with stem cells elsewhere, outside of Brazil, have indicated a relative improvement, but without any conclusive finding. These are studies undertaken without control groups and with patients affected by different types of Ataxia, which make it difficult to validate the research for specific groups in contexts such as Brazil (Saute and Jardim 2015). Most of them were conducted in countries, such as China and Thailand, with more flexible regulations concerning research and therapeutic use of stem cells and which promote so called ‘stem cell tourism’ (Murdoch and Scott 2010). It is notable that in 2015, a Brazilian lawyer with MJD successfully litigated against the Brazilian government to demand that they pay for his treatment with stem cells in Thailand.^15.^ Based on this decision, in March 2016, a patient with Friedreich's Ataxia managed to reverse a judicial decision which had originally denied her demand to a similar treatment, which will now also be paid by the Brazilian government.^16.^

Nevertheless, this kind of demand is not common among MJD patients in Brazil, where there is also no active mobilisation of patients’ organisations for the development of clinical research to find a cure for the disease or to access predictive tests. The main concern of Ataxia patients in general, and specifically of MJD carriers, is focused on minimising the effects of the disease, and the creation of specific clinical protocols to attend to their health care needs.

One avenue for achieving these aims for those affected has been to frame health care needs through approaches developed previously by those with disabilities. According to Monsores (2013), the increasing attention to the treatment of rare diseases in SUS was indirectly linked to the National Health Policy for Disabled People. This policy was created in 1989, and among its many guidelines was the stated responsibility of State to provide access to detect genetic diseases that can cause disability and offer genetic counselling to families. However, at that time it was not possible to achieve these objectives as there were insufficient genetic counsellors and medical geneticists to adequately structure and implement the policy.

Nevertheless, it is notable that recent government actions related to rights of disabled people are increasingly being encompassed also with reference to rare diseases. Recently, the Parliamentary Commission of Defense of the Rights of Disability People, approved a project that equates another disease, neurofibromatosis, to other physical and intellectual disabilities. The project will be examined by other commissions of Parliament and, if approved, will ensure the same social rights to people affected by neurofibromatosis as those who are disabled.^17.^ In March 2016, a new Parliamentary Front focused on Rare Diseases in the Chamber of Deputies was announced aiming to promote political debate about people with rare diseases, and construct an agenda to guarantee their access to health services, exams and diagnosis.^18.^

While these developments suggest that the association between ‘rare diseases/disability’ to promote ‘right to health’ and ‘social inclusion’ is automatic, this is far from being the case. More often, this has to be assembled between several actors as part of an uneven negotiation between patients’ organisations, politicians and health professionals. However, the link between disability rights and genetic research linked to rare diseases is particularly visible, in relation to diseases that affect children. Some researchers go so far as to defend actions to control rare diseases as a way to reduce the number of those with deficiencies among the population (Santos et al. 2013). While also raising the problematic issue of illegal abortion in cases of malformation or congenital anomalies, without other therapeutic options, genetic counselling and predictive tests are often seen as the main avenues for reaching this goal (Schuler-Faccini et al. 2014).

Nonetheless, some studies suggest that there is also a reluctance to undertake genetic testing or even receive genetic counselling, especially among asymptomatic subjects in families of those with MJD and other neurodegenerative diseases (Aureliano 2015; Osada 2012). In Brazil, this reluctance must be understood in relation to a complex set of variables linked to limited access to genetic tests in the public health system, the lack of therapeutic options to treat diseases identified by testing, the prohibition of abortion in cases of congenital abnormalities, the high cost of assisted reproduction techniques to select embryos (e.g. PGD Pre-Implantation Genetic Diagnosis) and the particular moral or religious restrictions related to contraception, among certain communities. In contrast to some hereditary cancers, notably those associated with the BRCA genes, there are additionally no preventive or prophylactic options for asymptomatic individuals undertaking predictive genetic testing for MJD.

In her research with families affected by MJD in Rio de Janeiro, Aureliano (2015) observed that people seek genetic testing when they start to experience the first symptoms of disease and also when they are seeking to justify leave from work or to access disability benefits. Two frequent symptoms of MJD are an imbalanced gait and slurred speech, and as a result many carriers are mistakenly taken to be drunk. Here, genetic tests also become a way to avoid this stigmatised identification, especially for men in their work environments. As one patient put it,

Had I said before [the test when the symptoms were milder] they would have said I was shirking, because I didn't look like I had any kind of disease […] Had I gone about telling everyone I had the disease, I would have been labelled a shirker (M., 54, retired after five years living with symptoms)

Asymptomatic relatives were also not keen to obtain predictive tests. This was often because of the lack of a cure or because it was felt positive results might negatively affect the person, provoking depression and anxiety, which it was believed might itself lead to early onset of MJD.

In our family we are only opposed to ‘this testing thing’. We know that for those that have this disease it's like an electronic device that comes out of the factory with a defect and is irreparable. If there is no cure, no treatment, why test before? (A., 64, asymptomatic, married with a cousin affected by disease, mother of two adult men, grandmother of two girls, none of them undertook predictive tests).

Reproductive planning based on genetic tests was not a concern to members of a number of families Aureliano investigated. However, other strategies were used to minimise risks or the appearance of disease in adult life, as these comments suggest.

I always thought that I had to have a job first [before getting pregnant], a good job, that would allow me to live, and if the disease appeared, one that would enable me to pay for a health plan for me and my child. (S., 37, asymptomatic, not tested, trying to get pregnant. Her mother and uncle have MJD).

Most rare diseases cause some kind of physical and/or intellectual impairment. Taking this into account, the association between ‘rareness’ and ‘disability’ appears as a justifiable strategy to get health access and other rights. However, this association is not immediate or consistent as the evidence demonstrated here suggests. In the case of MJD, for example, it becomes difficult to measure the level of handicap because the disease is unevenly progressive. The development of symptoms may be gradual in the first years, which makes it difficult to categorise some limitations as deficiency (for example, slurred speech). The heterogeneous phenotype of MJD can restrict access to certain rights guaranteed for disabled people, this includes, for example, special benefits to be paid in retirement in Brazil, whose value differs according to the level of handicap of the person.

On the other hand, in face of limited therapeutic options, the carriers of hereditary Ataxias in general have found in the policies and political attention to disabled people an avenue for social and political inclusion. Health professionals working with MJD patients have also mobilised around disability as an important means to offer more regular health care to their patients and to make it easier to access social rights.

Despite this, the ‘rareness’ of Ataxias is still the category and description normally used to frame the condition for patients and researchers, rather than its direct association with disability. In her research, Aureliano rarely observed those affected by MJD defining themselves as disabled, or affirming that there was a ‘deficiency’ in their family. Rather, they would frequently state that they carried or that their family had a rare disease. Nonetheless, her interlocutors demanded and used many social rights linked to disability, such as the electronic card that offers free use of urban buses.

This suggests that both categories, ‘rare’ and ‘disabilities’ are extended and limited to accommodate the specificities and variability of rare genetic diseases, thereby constituting various domains of inclusion and exclusion in the context of health care policy, politics and scientific research in Brazil. If the field of rare diseases offers possibilities for the future, with particular potential for innovation linked to genomics, the field of disability connects with the actual day to day realities of those affected by such conditions. Here, the capacity to offer care today is foregrounded by patients and also sometimes by professionals, rather than only the hope for a cure, which remains currently unknown and uncertain.

## Conclusion

In this article, we have examined how an emerging focus on rare genetic disease in Brazil is subject to and a product of particular dynamics of inclusion and exclusion at the interface with an expanding terrain of globalising genomic research and medicine.

In Brazil, a focus on rare genetic disease has emerged alongside the fledgling speciality of genetic medicine in close and complex relation with the goals of research and public health. This blurred boundary between research and clinical care has facilitated, but is now also challenged, by the growth of health judicialisation in Brazil; something which the recent national strategy to integrate rare genetic disease into the SUS public health system aims to resolve. In a context, where the state needs to manage limited resources to allow universal access to health care, as well as recognise the specificity of genetic diseases whose aetiologies and population frequencies are often contested or unknown, rareness can become a means to promote social justice and health care rights. The expanding terrain of judicialisation in Brazil makes this very evident. Yet, we have also seen how an association with rareness can lead to different sorts of potential or real exclusions, as entailed by the ethical responsibilities for treatment in the context of clinical trials in Brazil. While we recognise that this is an arena of development in which a range of different social stratifications inform the ability to leverage access to health care, partly in relation to income, social class and education, we argue that the ‘politics of difference’ at stake in Brazil stands in contrast to the politics of ‘inclusion’ as described by Epstein (2007) for the US. While in the latter, race and gender appear to more explicitly shape this process, in Brazil this is also informed by a politics predicated on efforts by patients, families and patient support organisations to oblige the state to meet a constitutional commitment to provide health care to all.

In the case studies we have explored of cancer genetics and Ataxias in Brazil, there are contradictory yet also productive movements that serve to both stabilise and destabilise research and medical practice related to these conditions within the parameters of ‘rare’ genetic disease. In the case of cancer genetics, we see how the pursuit of research and medical diagnosis related to the BRCA genes or R337h can be both enabled through an association with ‘rare’ cancer syndromes such as Li-Fraumeni or ‘unknown’ genetic variants in the case of BRCA genes. But this is also achieved through situated association with reference to the ‘frequency’ of the biomarker R337h in the south of the country or common cancers such as breast cancer. While in some cases an association with cancer as a common disease might lead to novel possibilities in terms of judicialised claims by certain enabled publics in the case of the BRCA genes, there are (at least currently) also limits to such possibilities in the case of R337h. In the case of Ataxia in Brazil, we see how there is a different kind of movement among researchers, patients and families between constituting the disease as ‘rare’ and/or associated with a wider class of disabling conditions in various situated contexts. The legitimacy of rights to health care resources and services (not always or necessarily cures, diagnosis or treatment, but also services and support) are made evident here as those living with conditions such as Ataxia or providing care for them negotiate different terrains through which mostly basic health care needs can be met.

By drawing attention to the parallel and contrasting movements in the dynamics between inclusion and exclusion in the case of cancer genetics and Ataxia in Brazil, we have shown how rare disease in the globalisation of genomic medicine is locally constituted and remade.

## Notes

1. https://www.gov.uk/government/uploads/system/uploads/attachment_data/file/210829/CMO_Science_priorities_letter_with_annexes.pdf (Accessed 16 November 2017).

2. Translational medicine or research (as distinct to ‘transnational’) aims to ‘translate’ basic or fundamental science research into medical practices and health outcomes.

3. We are grateful to the anonymous reviewer for highlighting the limits of ‘patient organisations’ as an analytical category particularly in relation to rare genetic disease in Brazil where activism may be ‘family orientated’ (see Fonseca 2015)and individualised models of patient activism may not be relevant.

4. In both cases, the majority of participants lived in urban regions (although not exclusively in Gibbon's research) and came from working and middle class backgrounds.

5. Judicialisation refers to the increased use of courts and judicial means for addressing public policy, including access to health resources. See page 5 for further discussion

6. In Brazil, this policy is popularly known as *teste do pezinho* (or the ‘blood spot test’), using samples collected from the heel of the newborn.

7. http://bvsms.saude.gov.br/bvs/saudelegis/gm/2014/prt0199_30_01_2014.html (Accessed on 20 April 2014).

8. Most of commentaries analysed by Castro (2015) came from families and individuals affected by Mucopolysaccharidosis IV-A (MPS IV-A) demanding the right to participate in international clinical trials.

9. See also Petryna 2009 for further discussion.

10. These are not by any means the only diseases of concern in medical genetics in Brazil, nevertheless their prioritisation themselves reflects processes of inclusion and exclusions. Due to public health screening programmes, conditions that appear in early life are prioritised. Equally conditions where there are treatment interventions, such as some cancers or metabolic conditions, are also a focus of interest in Brazilian medical genetics.

11. This is a possibility that has increased since 2013 following legislation to ensure that genetic testing for the BRCA genes could be included within the remit of private health insurance agreements or *convenios.*

12. ‘Care’ in return for samples is clearly controversial (see Sleeboom-Faulkner 2006) generating many ethical dilemmas in developing country contexts (see Traore et al. 2015)

13. For more information on the UFGRS Neurogenetic Network, see http://www.ufrgs.br/redeneurogenetica/ (Accessed 9 December 2014).

14. http://www.inagemp.bio.br/.

15. http://www1.folha.uol.com.br/cotidiano/2016/03/1751966-brasileiros-acionam-a-justica-por-tratamento-na-tailandia.shtml (Accessed 21 March 2016).

16. http://g1.globo.com/pa/santarem-regiao/noticia/2016/03/justica-bloqueia-r-130-mil-da-uniao-para-tratar-paraense-com-doenca-rara.html (Accessed 21 March 2016).

17. https://issuu.com/altacomplexidade/docs/pol__tica___sa__de_ed_042016_alt (Accessed 21 March 2016).

18. https://issuu.com/altacomplexidade/docs/pol__tica___sa__de_ed_052016 (Accessed 25 March 2016).
